# Advancing HEIs’ third-mission through dynamic capabilities: the role of leadership and agreement on vision and goals

**DOI:** 10.1007/s10961-021-09850-9

**Published:** 2021-04-04

**Authors:** Audrey Stolze, Klaus Sailer

**Affiliations:** 1 Munich University of Applied Sciences, Hess-Strasse 89, 80797 Munich, Germany; 2 Department of Business Start-Ups and Entrepreneurship, University of Hohenheim, Wollgrassweg 49, 70599 Stuttgart, Germany

**Keywords:** Entrepreneurial universities, Third mission, Strategic management, Dynamic capabilities, Leadership, Vision and goals, I23, O30

## Abstract

Higher education institutions (HEIs), once considered among society’s most resilient institutions, are facing challenges due to changes in governments’ and society’s expectations of them. Within the sector, there is a global call for new models and practices, requiring HEIs to develop the management capabilities once reserved for businesses. In this sense, they will pave entrepreneurial pathways and contribute to economic, technological and societal developments in their regions, thus adding a third mission (engaging socio-economic needs and market demands) to the traditional two (education and research) and transforming themselves into more entrepreneurial institutions. Dynamic capabilities enable transformation processes by allowing the dynamic sensing and seizing of opportunities and risks and the promotion of iterative change and reconfiguration. Scholars have called on HEIs to develop such dynamic capabilities in order to transform themselves and better respond to their sector’s challenges. Nevertheless, the understanding of how dynamic capabilities might advance HEIs’ third mission is still an underexplored concept, and in this paper, we propose mechanisms that promise to transform dynamic capabilities into third mission advancement. We have developed numerous theoretically grounded hypotheses and tested them with a partial least squares structural equation model into which we funnelled data collected from key decision-makers at German HEIs. The results suggest that dynamic capabilities do indeed influence third mission advancement; however, this relationship is mediated by the role of leadership and organisational agreement on vision and goals.

## Introduction

Even though higher education institutions (HEIs) may be among the most resilient and enduring institutions (Maassen and Stensaker 2011; Audretsch 2014), governments’ and society’s expectations of their contributions have evolved beyond the traditional roles of teaching and research. Now, the new norm in science is the capitalization of knowledge through a spiral model of innovation named Triple Helix, encompassing academia, government and industry in a transformative collaboration (Etzkowitz and Leydesdorff 1998). In this context, HEIs have been given a third mission: to actively contribute to economic, technological and social advancements by producing human, social and entrepreneurial capital (Etzkowitz et al. 2000; Guerrero et al. 2015). Higher education reforms have resulted in structural institutional changes (Maassen and Stensaker 2011) in which HEIs must demonstrate the ability to transform and evolve. Institutions that incorporate the third mission in this process are considered entrepreneurial (Etzkowitz 2004; Guerrero and Urbano 2012). Within this scenario, HEIs’ traditional management practices are no longer suitable (Teece 2018) and they therefore require new models for producing strategic advancements. Thus, the identification of entrepreneurial pathways for HEIs, which regard necessary strategic choices, are a key research agenda for the phenomenon of entrepreneurial HEIs (Klofsten et al. 2019).

Dynamic capabilities (DCs) are an essential concept in strategic management practices. They refer to an organisation’s ability to sense and seize opportunities, in order to reconfigure and transform itself, and are especially key in rapidly changing sectors. Thus, DCs enable value creation and the development of competitive advantages (Teece et al. 1997; Teece 2007; Wilden et al. 2013).

Previous research has pointed out that modern HEIs can be characterised as organisations that blend managerial practices and collegial professional values (Seeber et al. 2015), and the ideal of HEIs becoming more entrepreneurial is to be studied as a complex and multifaceted phenomenon (Kaša et al. 2019). Regarding DCs in higher education, studies have shown that they create value in universities’ technology transfer processes (Yuan et al. 2018), which is a key third mission activity. Overall, DCs provide HEI leaders with guidance in generating organisational adaptation (Leih and Teece 2016). These adaptions transpire via long iterative processes that are constantly influenced by exogenous and endogenous forces. Hence, such adaption processes require that DCs enable HEIs to develop new projects as experiments that sensitise stakeholders to the third mission so that it can be institutionalised (Stolze 2021).

Nevertheless, how DCs can support the strategic advancement of different types of organisations still requires further research (Vogel and Güttel 2013). In this context, scholars’ comprehensive understanding of how DCs facilitate HEIs’ third mission advancement is an important, but underexplored aspect. Against this background, this study addresses the research question of how can DCs be translated into HEIs’ strategic third mission advancements?

We answered this question using a research model that explored how third mission advancements in German HEIs occur by employing DCs through two routes: (1) leadership and (2) the establishment of a vision and goals. We took this approach because prior research suggested that developing strong DCs might require entrepreneurial leadership (Schoemaker et al. 2018) and an entrepreneurial vision (Wakkee et al. 2019).

We tested our theoretical model from explanatory and predictive perspectives using survey data from German academics who drive their institution’s third mission initiatives. The resulting measurement and structural models presented satisfactory outputs. We concluded that DCs alone have limited explanatory power in third mission advancement. A change-embracing leadership that effectively establishes a vision and goals through collaborative means mediates third mission advancements. Given this, our study’s contributions are threefold: (1) it further explains the relationship between DCs and HEIs’ third mission; (2) it identifies two mechanisms for effectively transforming DCs into third mission advancement; and (3) it offers managerial insights HEIs’ decision-makers can draw on to advance their institution’s third mission.

This article is structured as follows: first, we provide a theoretical foundation for our conceptual model and hypotheses. Then, we contextualise our research setting and explain our procedures before presenting and assessing the measurement and structural models’ results. After, we discuss this study’s implications and limitations to propose possible research avenues and render a conclusion.

## Theoretical framework and research model

### HEIs’ third mission and the triple helix

In the last three decades, many countries have reformed their higher educational systems, changing HEIs’ autonomy, public financing, mission and accountability. In Europe, for example, European Union directives and national government initiatives concomitantly affect HEIs (Curaj et al. 2018). Governments and societies’ expectations of HEIs have come to include more than teaching and research. Now, they are expected to be catalysts for regional economic, social and cultural development with the ultimate purpose of ensuring societies thrive’ in their entrepreneurial endeavours (Audretsch 2014). Thus, governments developed funding programmes to promote HEIs’ entrepreneurialism. Take, for instance, the British Science Enterprise Challenge, Dutch centres of excellence, the German EXIST or the Austrian A + B schemes (Mcgowan et al. 2008).

HEIs’ third mission can be seen as a second academic revolution (Etzkowitz 2003) in which enterprise is added to the traditional missions of teaching and research. Enterprising endeavours produce entrepreneurial capital and positively impact regional economies (Guerrero et al. 2015). HEIs that effectively incorporate the third mission are seen as entrepreneurial universities—a new paradigm introduced by Etzkowitz (1983) based on strategic developments at Stanford and the Massachusetts Institute of Technology (MIT) and their interactions with regional external stakeholders from the public and private sectors since their foundation in the late nineteenth century. The developments at these HEIs also influenced the conceptualization of the Triple Helix model proposition (Etzkowitz and Leydesdorff 2000).

Initially considered institutional anomalies because they deviated from the research university model (Etzkowitz 2004), Stanford and MIT now epitomise the entrepreneurial university ideal, inspiring HEIs around the world to emulate their achievements and attempt to build their own silicon valleys (Andersson et al. 2004; Etzkowitz 2019). In this sense, the Triple Helix model of university-industry-government interactions is a cornerstone for the development of emerging industries and new technology platforms, supported by governmental funding policies for basic and applied research, with potential to develop silicon valleys across the world (Etzkowitz 2015).

Managing HEIs’ advancement towards the third mission is more complex than one might think. In comparison to the average firm, within its Triple Helix interactions, an HEI has a broader range of stakeholders and a wave of heated and impactful political influences (Teece 2018). Research collaboration between industry and HEIs face overwhelming barriers regarding intellectual property, being this reduced when the rights appropriation is still uncertain and the research less public, reducing tensions between actors (Hall et al. 2001). In this sense, HEIs’ technology transfer performance depends on building trustful relationships among regional actors and implementing flexible institutional policies towards patenting, licensing and intellectual property rights (Santoro and Gopalakrishnan 2001).

Hence, HEIs’ governance and leadership style play a key role in the success—or failure—of strategically advancing the third mission (Garcia et al. 2012). For instance, the case of the University of Bari in Italy demonstrates that the third mission is mainly enabled by ‘an open model of governance with internal and external stakeholder involvement’ (Lombardi et al. 2019, p. 3394).

As an influential sphere in the Triple Helix, governments have pushed HEIs to make changes in their governance structure so they can be ‘more effective, efficient and responsive to societal needs’ (Capano and Pritoni 2020, p. 2), providing the necessary support for entrepreneurship and related education (Guerrero et al. 2011). Thus, propositions to transform HEIs into entrepreneurial universities include governance and leadership as key drivers, which was already reflected in Clark's (1998) strengthened steering core proposition and Nelles and Vorley's (2011) entrepreneurial blueprint.

### HEIs’ leadership and the establishment of visions and goals

In HEIs, leadership must incorporate a collegiality ethos into management approaches, as this is critical in order for change management processes to ‘create vision, communicate policy and deploy strategy’ (Davies et al. 2001, p. 1026). When proper leadership is missing, an institution is seen as hindering its own development and performance, as in the case of some African HEIs (Muriisa 2014). Furthermore, the ‘relationship between government and universities implies a “black-boxing” of academic leadership’ (Ekman et al. 2018) of which we still know little about.

HEIs’ presidents, provosts and chancellors shape their institution’s developmental path (Eddy and Vanderlinden 2006). The strong leadership provided by these individuals support HEIs’ transformation into more entrepreneurial universities (Yokoyama 2006; Wakkee et al. 2019). Cases illustrating advances in HEIs’ third mission have highlighted the key roles chief executives play, including at Stanford (Etzkowitz 2003; Leih and Teece 2016), MIT (O’Shea et al. 2007) and Garfield State (Mcclure 2016) in the United States; further cases have been made of the Chalmers Institute of Technology in Sweden (Jacob et al. 2003; Berggren 2011) and the University of Itajubá in Brazil (Almeida 2008). Hence, HEIs’ senior management support is essential, as these people hold ‘sufficient managerial authority to be able to make decisions in the process of consultation and to convince sophisticated individuals that the transition would have a beneficial effect’ (Mcroy and Gibbs 2009, p. 697). In order to promote transformative organisational change, HEIs’ leaders must obtain support from the broader academic community (van Ameijde et al. 2009) and include external stakeholders (Etzkowitz and Leydesdorff 1998) in an environment of co-creation (Mader et al. 2013).

In this context, clear communication between HEIs’ leaders and its scholars and staff is essential, as it influences the organisational climate and the ‘faculty’s intellectual leadership behaviours’ (Uslu and Arslan 2018, p. 408). Effective communication is fundamental in empowering individuals and managing the internal politics related to, for instance, the distribution of funds for third mission initiatives (Garcia et al. 2012). A key element of this communication is institutional vision, as HEIs must re-envision themselves to produce change (Hamington and Ramaley 2018), set goals and establish an entrepreneurial vision to enable their transformation into more entrepreneurial entities (Wakkee et al. 2019). Thus, public institutions should focus on developing a shared vision and its implementation (Volcker 2014). Additionally, clearly defined goals have been identified as enablers of the emergence of effective distributed leadership in HEIs (van Ameijde et al. 2009), and as HEIs and industries have different goals, clear defined goals are a pre-requisite for the successful completion of technology transfer initiatives (Hidalgo and Albors 2011).

According to Battilana et al. (2009), developing a vision in an institutional context requires mobilising allies and motivating stakeholders to achieve and sustain it. HEIs’ strategic planning activities rely on a vision, and the process of its development must be participative (Özdem 2011). However, the actual role and effect of a vision on HEIs’ performance is not yet well researched (Kantabutra 2010), which leaves a gap in the understanding of its effect on strategic advancement.

### DCs and their role in HEIs

DCs are a conceptual proposition introduced by Teece et al. (1990) and refer to an organisation’s ability to sense and seize opportunities and threats in order to strategically promote change. Sensing means monitoring and identifying signs of possible change, even if weak, in the organisation’s meso and macro environments. In order to sense, the organisation must establish an analytical system supported by individuals’ ability to learn and sense in order to filter, shape, and calibrate opportunities (Teece 2007). At the same time, effectively sensing threats enables an organisation to mitigate the associated risks.

Effectively sensing opportunities allows an organization to seize them through timely innovations that increase its competitive advantage, through the development and launch of new processes, products and services. According to Teece (2007) ‘Addressing opportunities involves maintaining and improving technological competences and complementary assets and then, when the opportunity is ripe, investing heavily in the particular technologies and designs most likely to achieve marketplace acceptance’.

However, in volatile environments, sensing and seizing are not enough to produce effective responses, requiring organisations to reconfigure and constantly adapt to change. An organisational reconfiguration can refer to its structures, processes, designs and incentive schemes (Teece 2007). To develop strong DCs, organisations need entrepreneurial leadership, as this process requires more experimentation than detailed planning (Schoemaker et al. 2018). Simply said, it requires more entrepreneurialism and less management.

The concept of DCs borrows and combines elements from strategic management, evolutionary economics and behavioural theory (Vogel and Güttel 2013) to explain how organisations leverage their capabilities to respond to swift environmental changes and create new competitive advantages (Teece et al. 1997). Since the 1990s, the concept has gained momentum among researchers but still remains a novel proposition requiring a stronger foundation of empirical studies regarding antecedents, mechanisms (moderators and mediators) and consequences, potentially with process-based approaches to DCs evolution (Schilke et al. 2018) and how these may support the strategic advancement in different types of organisations (Vogel and Güttel 2013).

In the context of HEIs, DCs are considered a key micro-foundation element of HEIs’ intrapreneurial capabilities (Guerrero et al. 2020). Strong DCs are able to create value for different internal and external stakeholder groups while at the same time protecting the academic ethos (Siegel and Leih 2018; Teece 2018). For instance, Stanford’s successful strategic advancements towards the third mission and recognition as epitomising the entrepreneurial university model has been attributed to its superior dynamic capabilities (Leih and Teece 2016) in comparison to other institutions. Furthermore, Leih and Teece (2016) also proposed that HEIs leaders’ DCs positively influence work commitment, ultimately contributing to HEIs’ performance. Here, the question remains as to what extent and how DCs contribute to HEIs’ third mission advancement.

## Research model and hypotheses

**Fig. 1:**
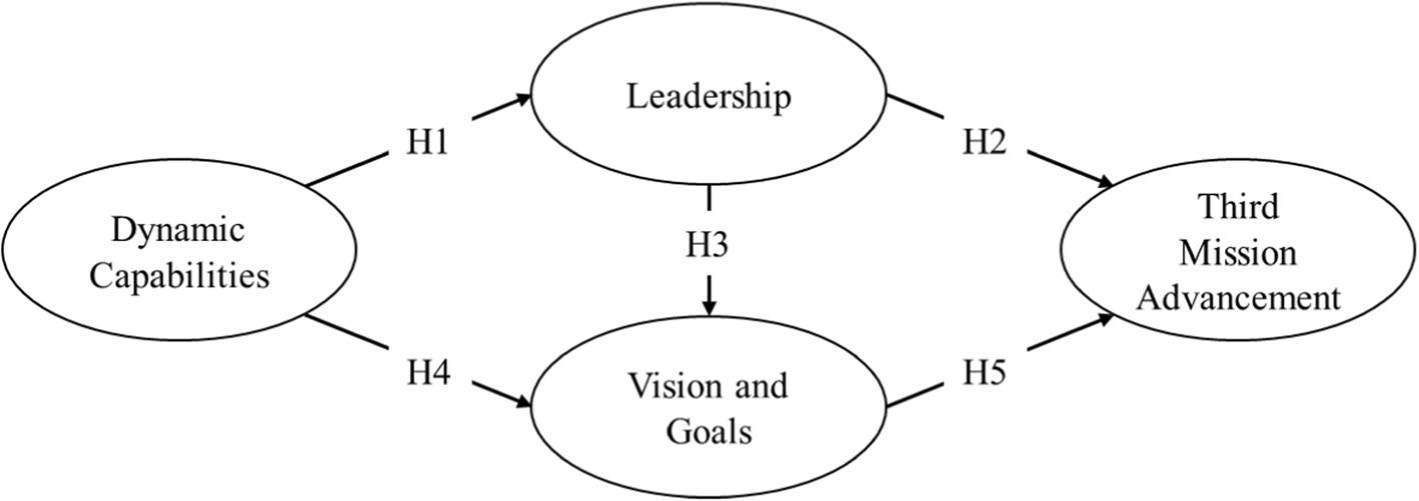
Conceptual model

Our proposed research model (Fig. 1) illustrates our hypotheses and allowed us to investigate to what extent leadership and agreement on vision and goals provide effective routes that enable DCs to assist third mission strategic advancement. We assumed that leadership and agreement on visions and goals mediate DCs impact on third mission advancement, theorising that an HEI with strong DCs can provide the necessary leadership to reach agreements on vision and goals, enabling greater flexibility and a multitude of entrepreneurial pathways to the advancement of its third mission.

Based on the theory reviewed, we conceptualised two routes composed of five hypotheses (Fig. 1). The first hypothesis stated that *DCs are positively associated with the leadership of an HEI’s governing body* (H1). This hypothesis built on three facts: first, leadership is required to incorporate an ethos of collegiality into management practices (Davies et al. 2001); second, entrepreneurial leadership is required to develop strong DCs (Schoemaker et al. 2018); and third, DCs produce value for different stakeholders while protecting an academic ethos (Siegel and Leih 2018; Teece 2018).

Additionally, strong leadership supports HEIs’ transformation into more entrepreneurial universities (Yokoyama 2006; Wakkee et al. 2019), and many institutional cases across the world illustrate this in the literature (e.g. Stanford, MIT, Itajubá and Chalmers). These leaders’ management styles influence the success or failure of third mission strategic advancement (Garcia et al. 2012). This happens because top managers have the authority to convince internal and external stakeholders to produce institutional change (Mcroy and Gibbs 2009). Hence, we assumed that the *leadership provided by an HEI’s governing body is positively associated with third mission advancement* (H2).

Moreover, due to the convincing power of leaders over ‘sophisticated individuals’ (Mcroy and Gibbs 2009, p. 697) who are part of different stakeholder groups within HEIs Triple Helix interactions, we also theorised that the *leadership provided by an HEI’s governing body is positively associated with agreement on its vision and goals* (H3). This is so for two reasons: first, in institutional contexts, the development of a new vision, achieving it and sustaining it require motivating all stakeholder groups and mobilising allies (Özdem 2011); second, clearly defined goals enable effective distributed leadership in HEIs (Garcia et al. 2012).

The formulation of a vision through participatory processes is fundamental to HEIs’ strategic planning (Özdem 2011). Given this and the fact that DCs are an essential concept in strategic management practices designed to produce change, our fourth hypothesis stated that an *HEI’s DCs are positively associated with organisational agreement on vision and goals* (H4). Moreover, on the grounds that to produce change and transformation HEIs need to first re-envision themselves (Hamington and Ramaley 2018) and that entrepreneurial visioning and goal setting enable their transformation into more entrepreneurial institutions (Wakkee et al. 2019), our fifth hypothesis was that *agreement on vision and goals is positively associated with third mission advancement* (H5).

## Methods

### Sample and data collection

We conducted a survey with key respondents from German HEIs to test our hypotheses using a structured online questionnaire. For the purpose of this survey, key respondents were defined as academics (professors, project managers or associate researchers) who were among the key people driving the third mission in their institutions. Specifically, we contacted the individual responsible for their institution’s successful application to EXIST-Potentiale conceptual and/or final phases (GFMEAE 2020), a recent federal government scheme aimed at progressing German HEIs’ third mission. The two-phased application process unfolded in 2019 and required HEIs to strategically conceptualise (concept phase) and pilot (final phase) third-mission-related initiatives that successful applicants shall implement in the near future. This scheme had three modules: (1) *Potentiale Heben* (‘Increase Potential’) targeted small- and medium-sized institutions that needed to (further) develop their third mission initiatives; (2) *Regional Vernetzen* (‘Connect Regionally’) targeted HEIs that aimed to (further) develop their regional entrepreneurial ecosystem; (3) and *International Überzeugen* (‘Promote Internationally’) focused on entrepreneurial universities that aimed to (further) internationalise their third mission.

The above context provided us with an up-to-date, qualified mailing list of key respondents who recently coordinated a large, institutional and strategic third mission planning process. The procedure allowed us to approach a diverse group of HEIs rather than focus on institutions already recognised as entrepreneurial universities (see “Appendix A”). This unique research setting was especially relevant to our study, as we aim to explain third mission advancements in HEIs, regardless of their current developmental stage.

In total, 201 distinct institutions were approved in the first conceptual phase and/or in the final phase of EXIST-Potentiale. From those, we contacted 194 HEIs, excluding seven medical schools / university hospitals. First, we conducted a pilot study at our own HEIs to pre-test the questionnaire. We implemented small changes regarding instructions and clarifications of the constructs. In April 2020, we electronically collected the data by sending all 194 respondents personalised invitations and up to two reminder e-mails to complete the online form. We obtained a 23% valid response rate (45 individuals) after excluding 28 incomplete questionnaires—a response-rate considered adequate for organisational studies with key respondents (Baruch and Holtom 2008). A characterisation of the sample, including HEI profiles, is available in “Appendix A”.

### Measures

This confirmatory study’s measures for further developing a theory on the effect of DCs on HEIs’ third mission was built on validated scales available in the literature. We adapted these to the context of HEIs based on the theoretical foundation available, and we operationalised all independent constructs into a 7-point Likert scale (1 = ‘strongly disagree’ to 7 = ‘strongly agree’). The dependent construct Third Mission Advancement was operationalised via two distinct semantic 5-point Likert-scales as a procedural remedy to mitigate common method bias (Podsakoff et al. 2003). The questionnaire was organised per construct and in blocks, offering the constructs’ descriptions to participants before the indicators they had to rate.

*DCs* As reflective constructs in explorative models are allowed redundancy, 14 indicators were adapted from Wilden et al. (2013) and Kump et al. (2018) borrowing concepts from two qualitative study on HEIs’ DCs (Leih and Teece 2016; Teece 2018)**.** During the calculation of the measurement model, we excluded five indicators due to redundancy, below-threshold reliability and/or discriminant validity (Hair et al. 2011). The nine remaining indicators loaded above 0.70 and are described in Table 1 (α = 0.912).

**Table 1:** Constructs’ validity and reliability and indicator factor loading and significance

Constructs	Factor loading	*t*-value*
DCs (Cronbach’s α = 0.912; rho_A = 0.925; CR = 0.927; AVE = 0.586)		
DC_1: ‘At my HEI, members participate in activities in the regional ecosystem’	0.731	6.229
DC_2: ‘At my HEI, we systematically monitor developments in the higher education sector in Germany’	0.831	8.616
DC_3: ‘At my HEI, we systematically monitor developments in the higher education sector abroad’	0.708	5.240
DC_4: ‘My HEI benchmarks the third mission initiatives of other German HEIs’	0.743	13.211
DC_5: ‘My HEI monitors the performance information of third mission initiatives’	0.816	18.401
DC_6: ‘My HEI invests to develop projects that solves regional ecosystem stakeholders’ problems’	0.703	5.608
DC_7: ‘My HEI adopts best practices for third mission initiatives’	0.856	21.672
DC_8: ‘At my HEI, we listen to the needs of regional ecosystem stakeholders and develop new projects accordingly’	0.732	5.272
DC_9: ‘At my HEI, we frequently change or adapt practices and processes based on feedback from internal and external stakeholders’	0.755	6.169
Leadership (Cronbach’s α = 0.943; rho_A = 0.944; CR = 0.951; AVE = 0.637)		
L_1: ‘My HEI’s senior leaders communicate and reinforce the institution’s entrepreneurial values’	0.790	7.531
L_2: ‘My HEI’s senior leaders provide personal leadership for third-mission-related projects’	0.768	6.399
L_3: ‘My HEI’s senior leaders create and communicate a vision focused on the third mission’	0.808	9.209
L_4: ‘My HEI’s senior leaders are personally involved in improvement of third-mission-related activities’	0.837	8.415
L_5: ‘My HEI’s senior leaders participate in the third-mission-related activities’	0.818	11.334
L_6: ‘My HEI’s senior leaders consider the improvement of third-mission-related activities a way to strategically advance the HEI’	0.753	10.243
L_7: ‘My HEI’s senior leaders view the third mission as being as important as the teaching and research missions’	0.807	12.910
L_8: ‘My HEI’s senior leaders allocate adequate resources to efforts related to the third mission’	0.790	17.329
L_9: ‘My HEI’s senior leaders repeatedly tell professors and staff that its advancement depends in it adapting to regional ecosystem stakeholder demands’	0.791	11.463
L_10: ‘My HEI’s senior leaders repeatedly tell professors and staff that building, maintaining and enhancing relationships with regional ecosystem stakeholders is critical to its advancement’	0.793	12.104
L_11: ‘My HEI’s senior leaders repeatedly tell professors and staff that collaborating and co-creating with regional ecosystem stakeholders is critical to its advancement’	0.821	15.176
Vision and Goals (Cronbach’s α = 0.847; rho_A = 0.854; CR = 0.898; AVE = 0.688)		
VG_1: ‘My HEI has common goals related to the third mission’	0.844	15.207
VG_2: ‘My HEI is actively involved in standardising third-mission-related practices and operations’	0.779	8.451
VG_3: ‘My HEI clearly cooperatively defines third-mission-related roles and responsibilities with internal stakeholders’	0.909	34.763
VG_4: ‘At my HEI, we all know which members are responsible for which third mission activities’	0.778	6.679
Third Mission Advancement (Cronbach’s α = 0.809; rho_A = 0.827; CR = 0.912; AVE = 0.839)		
TMA_1: Description that best fits the HEI’s third mission development status: (1) ‘My HEI has not yet started to develop or implement third-mission-related initiatives’; (2) ‘My HEI has started to develop third-mission-related initiatives but has not implemented them yet’; (3) ‘My HEI started to implement third-mission-related initiatives’; (4) ‘My HEI is currently consolidating third-mission-related initiatives’; (5) ‘My HEI has already institutionalised its third-mission-related initiatives.’	0.901	24.232
TMA_2: HEI third-mission performance in comparison to other German HEIs is: (1) ‘Insignificant’; (2) ‘Below average’; (3) ‘Average’; (4) ‘Above average’; (5) ‘We are one of the leading HEIs in the country’	0.931	33.651

*Significance level: 0.05

*Leadership* This construct was presented to the study’s participants in the following manner: ‘With the following items, we would like to assess how engaged your HEI’s senior leaders are in third-mission-related initiatives and future planning. Please consider your HEI’s president, vice-presidents and board(s) of governors as senior leadership (i.e., *Senate*; *Hochschulräte*).’ Drawing on validated scales measuring leadership (Ahire et al. 1996; Min and Mentzer 2004; Peng et al. 2008; Oliveira and Roth 2012), we conceptualised 19 indicators, and following the same assessment procedure conducted for the DC measures, we excluded eight items. All remaining indicators (Table 1) loaded above 0.70 (α = 0.943).

*Agreement on vision and goals* The four applied indicators were borrowed from Min and Mentzer’s (2004) validated scale. These were operationalised by adapting them to the context of this study (Table 1), and they were satisfactorily loaded above 0.70 (α = 0.847).

Third mission strategic advancement Before exploring this construct, we presented participants with an explanation of the third mission concept: ‘When answering this question and the remainder of the questionnaire, please take into consideration that higher education institutions’ (HEIs) third mission refers to an additional function of HEIs in the context of knowledge societies. For the purposes of this study, it includes a wide range of initiatives that aim to positively impact the development of HEIs’ regional ecosystems in economic, technological and societal terms.’ The lack of a suitable validated scale to assess this construct led us to conceptualise two semantic scales. First, regardless of an HEI’s stage of third mission development, we proposed a 5-point Likert scale. Our proposition discerned change strategy conceptualisation and implementation (Herrmann and Nadkarni 2014; Heyden et al. 2017) and was derived from a recent action framework proposed to make HEIs more entrepreneurial (Stolze 2021). The first indicator loaded at 0.901, and its five Likert points read: (1) ‘My HEI has not yet started to develop nor implement third-mission-related initiatives’; (2) ‘My HEI has started to develop third-mission-related initiatives but has not implemented them yet’; (3) ‘My HEI started to implement third-mission-related initiatives’; (4) ‘My HEI is currently consolidating third-mission-related initiatives’; and (5) ‘My HEI has already institutionalised its third-mission-related initiatives.’ The second indicator took into consideration the intensifying competition in the higher education sector (Brankovic 2018; Klofsten et al. 2019) to asses competitive performance and borrowed from Mikalef and Pateli (2017). This indicator rated HEIs’ third mission performance in comparison to other German HEIs as: (1) ‘Insignificant’; (2) ‘Below average’; (3) ‘Average’; (4) ‘Above average’; or (5) ‘We are one of the leading HEIs in the country.’ This indicator loaded at 0.931, and this novel construct conceptualisation proved to be a reliable proposition (α = 0809).

*Common method bias control* Self-report questionnaires are a well-known problem in organisational research, and the challenges they introduce need to be adequately addressed (Podsakoff and Organ 1986). Therefore, we employed the procedural remedy of having different response formats (Podsakoff et al. 2003). The dependent construct (Third Mission Advancement) was measured via two distinct semantic 5-point Likert scales, while the independent variables were measured with a standard 7-point agreement Likert scale. Moreover, we structured the questionnaire in blocks, one per construct, and provided adequate descriptions.

## Results

### Measurement model assessment

We employed the variance-based structure equation modelling technique partial least squares path modelling (PLS-SEM) to assess our measures and test our hypothesised model with support from the software SmartPLS3 (Ringle et al. 2015). PLS-SEM is considered a robust yet flexible technique suitable in diverse situations (Hair et al. 2011, 2012), and it is widely employed in management research and increasingly in higher education studies (Ghasemy et al. 2020). It is a particularly suitable technique in estimations of complex causal predictive models with more parameters than observations or when observations are restricted by small populations, as it computes measurement and structural model relationships separately instead of simultaneously (Hair et al. 2019). Given that our sample was technically small but could not be reasonably extended because of the limited overall population of German HEIs, PLS-SEM was an appropriate approach. In order to provide concise and precise reporting, we followed state-of-the-art procedural guidelines offered by Hair et al. (2019) and Ghasemy et al. (2020).

First, we examined the indicators’ factor loading. All indicators loaded above 0.70 (Table 1). A recent recommendation suggested a threshold of 0.708 for loadings—up from the widely applied 0.60 threshold—meaning the construct explained more than 50% of its indicator’s variance (Hair et al. 2019). Only one indicator (DC_6) loaded slightly below this more conservative threshold at 0.703.

Next, we assessed the constructs’ internal consistency reliability via three distinct methods recommended by Hair et al. (2019): (1) composite reliability, which provides the highest results, as items are weighted; (2) Cronbach’s alpha, a more conservative unweighted measure; and (3) rho_A, an intermediate measure proposed as a more precise construct reliability measure (Dijkstra and Henseler 2015). All our constructs presented good reliability based on these measurements, since they were far above the satisfactory threshold of 0.70 (Table 1).

Next, we assessed convergent validity and discriminant validity. First, on the construct level, we checked for average variance extracted (AVE), which has a threshold of 0.50. All our constructs presented good convergent validity (Table 1). To verify discriminant validity, we checked the traditional Fornell-Larcker criterion (Table 2) and the novel Heterotrait-Monotrait ratio (Table 3); the latter is considered a reliable and more precise measurement in PLS-SEM (Franke and Sarstedt 2019). All constructs were empirically distinct from each other, since their shared variance was lower than their AVE (Fornell and Larcker 1981), and all had heterotrait-monotrait ratios below the maximum of 0.85 (Henseler et al. 2015; Franke and Sarstedt 2019). On the item level, we checked their factor loadings versus cross-loadings to assess discriminant validity (“Appendix B”). All items loaded the highest on their respective constructs, confirming the indicators’ discriminant validity.

**Table 2:** Constructs’ Fornell–Larcker criteria

	Third mission advancement	DCs	Leadership	Vision and goals
Third mission advancement	0.916			
DCs	0.559	0.766		
Leadership	0.653	0.679	0.798	
Vision and goals	0.669	0.735	0.662	0.829

**Table 3:** Constructs Heterotrait–Monotrait ratios

	Third mission advancement	DCs	Leadership	Vision and goals
Third mission advancement				
DCs	0.617			
Leadership	0.733	0.704		
Vision and goals	0.808	0.790	0.729	

Last, we examined collinearity to assure it did not result in biased regression results (Hair et al. 2019), a check recommended in PLS-SEM studies (Kock 2015). The accepted threshold for this check is a variance inflation factor of 3.3. However, as PLS-SEM algorithms effectively reduce model-wide collinearity, a higher threshold (5 or even 10) may also be acceptable (Kock and Lynn 2012). Our model’s constructs did not present collinearity issues (Table 4).

**Table 4:** Constructs collinearity statistics (variance inflation factor)

	Third mission advancement	DCs	Leadership	Vision and goals
Third mission advancement				
DCs	2.540		1.000	1.855
Leadership	2.078			1.855
Vision and goals	2.440			

### Structural model assessment

**Fig. 2:**
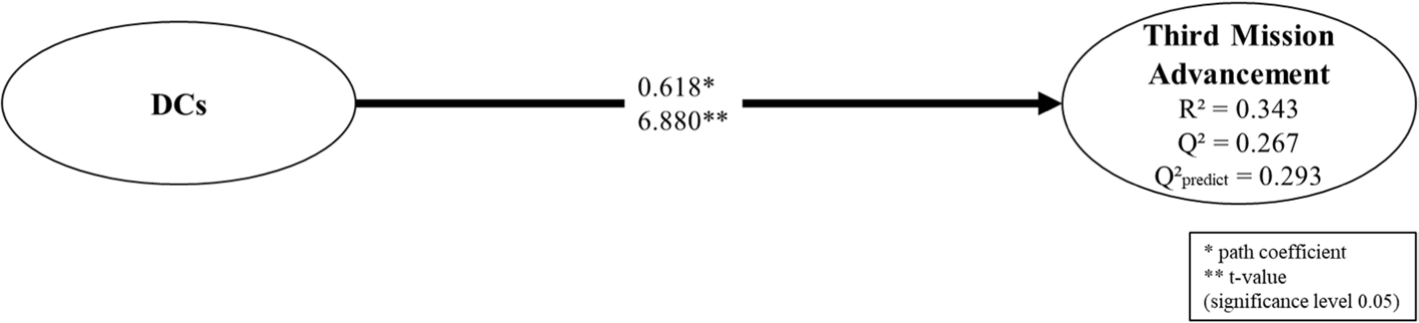
Direct model without mediation

**Fig. 3:**
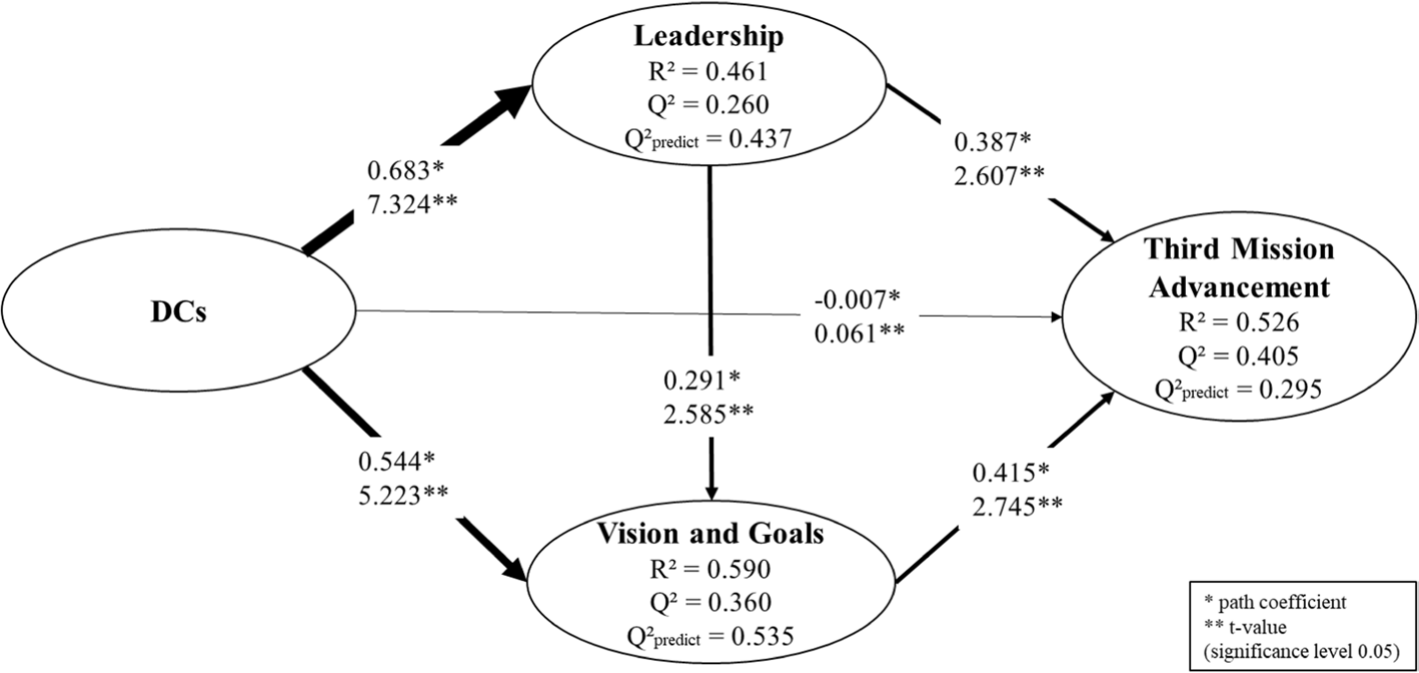
Proposed model with mediation

Before assessing our structural model, we produced a direct model without mediation (Fig. 2) to establish a benchmark for comparing results in order to complement our assessment of how DCs affect third mission advancement. The direct model proved to be valid, though it demonstrated lower explanatory power in comparison to our mediated model (Fig. 3), as its R^2^ was 0.343 versus 526. Nevertheless, it offered a very similar out-of-sample prediction power (Q^2^ predict = 0.293 vs. 295 in Figs. 2 and 3).

In order to assess our proposed structural model (Fig. 3), we first verified the coefficient of determination (R^2^), which expresses association level but not causation (Shmueli 2010), thus measuring the model’s explanatory power. According to methodological guidelines (Hair et al. 2011), our proposed model presented moderate explanatory power with R^2^ of 0.461 (Leadership), 0.526 (Third Mission Advancement) and 0.590 (Vision and Goals).

Next, we employed a blindfolding procedure to calculate the Q^2^ value, which combines in-sample explanatory power with out-of-sample prediction elements. Even though researchers routinely use this metric to assess a model’s predictive accuracy, recent methodological guidelines argued that it is imprecise because it is not an out-of-sample-only measurement (Shmueli et al. 2019). Therefore, in addition to reporting the Q^2^ value (Fig. 3), we calculated a recently developed prediction power measurement, namely PLS Predict (Q^2^ predict). With recommended setting (10 subsets; 10 repetitions), we observed (see “Appendix C”) that all indicators used to measure Third Mission Advancement and Vision and Goals presented via PLS were lower than what was obtained via a linear regression model, which is considered a ‘naïve’ benchmark (Shmueli et al. 2019, p. 2326). Therefore, the model had a high predictive power for these constructs. A medium predictive power was observed for leadership, as one of its indicators (L_11) had a slightly lower root mean square error caused by linear regression (Hair et al. 2019; Shmueli et al. 2019).

After confirming the explanation and prediction powers of our structural model, we assessed its paths significance by calculating their coefficients and *t*-values (Fig. 3). We ran the recommended two-tailed complete bootstrapping with 5000 subsamples at a significance level of 0.05 using the bias-corrected and accelerated bootstrap confidence interval method. This was the preferred procedure because confidence intervals could be adjusted for data ‘skewness’ (Hair et al. 2019, p. 6).

The size of path coefficients were aligned with the observed effect size (f^2^), making the reporting of the latter redundant (Hair et al. 2019). Based on the resulting t-values, all but one path (from DCs directly to third mission advancement) were relevant, with arrows’ widths illustrating their relative relevance (Fig. 3). Moreover, to assess the mediating effect of Leadership and Vision and Goals, we checked for the specific indirect effect of DCs on Third Mission Advancement (Nitzl et al. 2016). The results showed that the mediated paths were relevant (Table 5).

**Table 5:** Path-specific indirect effects

	Original sample	Sample mean	STDE	*T*-value	*P*-value
DCs → Leadership → Third mission advancement	0.257	0.261	0.112	2.293	0.022
DCs → Vision and goals → Third mission advancement	0.226	0.224	0.098	2.302	0.021
DCs → Leadership → Vision and goals	0.205	0.202	0.091	2.252	0.024

When compared to the results of the direct model (Fig. 2), the assessment of the mediated structural model confirmed that both theorised routes are valid and offer superior explanations to the relationship between DCs and third mission strategic advancement. Specifically, HEIs’ DCs are indeed positively associated with the leadership of its governing body (H1) and with agreement on vision and goals (H4), while the leadership provided by an HEI’s governing body is positively associated with organisational agreement on vision and goals (H3). Additionally, leadership provided by an HEI’s governing body and agreement on vision and goals are positively associated with an HEI’s third mission advancement (H2 and H5, respectively).

## Discussion

In this study, we examined how DCs facilitate third mission advancements in HEIs and assessed to what extent leadership and agreement on vision and goals provide effective routes that enable DCs to assist third mission advancements. We tested our hypotheses through a PLS-SEM analysis, as this method is particularly useful in predicting and identifying an outcome’s drivers (Hair et al. 2011, 2019). We surveyed key respondents from 45 German HEIs in different stages of pursuing entrepreneurial pathways. This was a key setting, as prior empirical research generally analysed successful cases retrospectively, potentially leading to biases and contextual findings (Battilana et al. 2009).

We measured third mission advancement based on the perceived development stage and national competitive performance. Our results confirm that DCs play in important role in facilitating such advancements in HEIs. Specifically, German HEIs’ ability to sense opportunities by benchmarking other German HEIs and monitoring their third mission initiatives are key capabilities. Sensing by benchmarking leads HEIs to adopt best practices in order to transform themselves into more entrepreneurial institutions. This strategy might be the result of a relatively late start to introducing third mission initiatives. However, there are dramatic limitations to emulation strategies due to differences in environmental context, resources and internal capabilities (Etzkowitz and Zhou 2008; Philpott et al. 2011; Stensaker and Benner 2013). Thus, German policy makers need to evaluate carefully the replication of foreign legislative instruments and its success measurement criteria to not generate unintended consequences, as when reforming the former German professor’s privilege influenced by the US Bayh-Dole Act to create in 2002 the German Employees’ Invention Act (Cunningham et al. 2019).

Teece (2018, p. 01) argued that HEIs require *‘*institutional introspection, cultural change and the development of effective processes for diagnosing problems and reaching decisions. Strong dynamic capabilities can help a university confront the uncertainty surrounding new technologies and prioritize resource allocation to favour the future.’ Our empirical analysis confirm his essay’s argumentation and builds on it by demonstrating the mediating role of leadership and agreement on vision and goals.

We also found that third-mission-related roles and responsibilities must be defined cooperatively among internal stakeholders in order to achieve an agreement on goals and develop a vision. For this to succeed, HEIs’ presidents and governing bodies must provide the necessary leadership by allocating adequate resources to efforts related to the third mission and telling professors and staff that they should build, maintain and enhance relationships with regional ecosystem stakeholders, as collaborating and co-creating with them is critical to HEIs’ advancement. In this sense, leaders must take into account that entrepreneurial ecosystem stakeholders’ preoccupations and interests regarding HEIs’ future roles produce normative scenarios driven by internationalisation, digital transformation, collaborative networks and co-creation processes (Stolze and Sailer 2020).

In light of this study’s results and discussion, its contributions are threefold. First, it further explains the relationship between DCs and HEIs’ third mission. It empirically confirms the relevance of DCs in advancing HEIs’ third mission by demonstrating that they are in fact influenced by the mediating role of leadership and agreement on vision and goals. Its second contribution is the identification and confirmation of two mechanisms through which DCs can be employed to enhance and predict third mission advancement. These two contributions were achieved following state-of-the-art application and reporting recommendations for PLS-SEM studies (Hair et al. 2019; Ghasemy et al. 2020), offering novice scholars a didactic example of the method’s use in higher education studies. Last, this study offers managerial insights HEIs’ decision-makers can draw on to advance their institution’s third mission.

### Management implications

Our discussion offers managerial insights into how HEI decision-makers advance their institutions’ third mission, as it further elaborates and exploits the critical role of governance already identified as a key entrepreneurial pathway (Stolze 2021). Our findings indicate that a prerequisite for this strategic change process is that HEI leaders consider the third mission as being as important as the teaching and research missions. Middlehurst (2013, p. 276) questioned if HEIs’ leaders are ‘fit for the future’, as institutional governance ‘is messy and contested territory where the boundaries between levels are blurred and where power and authority between different actors in the system are in flux’. In this sense, a recent resolution from the German Rectors Conference, made a call for German HEIS to face their challenges as ‘dynamic establishments attuned to change, […] responding to competition, continuously developing their structures and seeking dialogue with all important social groups’ (HRK 2018, p. 4). Henceforth, HEIs must pro-actively manage their Triple Helix interactions, taking into account entrepreneurial ecosystem stakeholders’ preoccupations and interests towards them, which result in foresights driven by internationalization, digital transformation and co-creation processes (Stolze and Sailer 2020). Thus, HEIs must co-create, co-fund and co-manage new formats that advance their third mission through Triple Helix interactions. At the same time, policy-makers must enable it through supportive policies, funding schemes and increased autonomy for HEIs to collaborate with external stakeholders.

Consequently, there should be a policy call for HEI leaders’ professional development to provide them with the necessary business skills and relationship management competences (Tran and Nghia 2020). Periodical participation in external training, mentoring and auditing schemes should become standard practice, as co-creation processes could be fundamental for HEIs to advance strategically their third mission. Our study indicates that German HEIs’ ability to sense opportunities is influenced by benchmarking and monitoring practices, external auditing schemes could support context-specific analysis for the development of tailored advancement strategies. Some example of initiatives supporting HEIs’ leaders in such endeavours are the international programs HEInnovate, UIIN (University-Industry Innovation Network) and the Triple Helix Association; and in Germany, the HRK (German Rector’s Conference) and the DenkFabrik.^1^

### Limitations and future research avenues

Some limitations of this study open interesting avenues for future research. First, our sample concentrates on German HEIs and hence includes the contextual singularities of that country’s higher education system and entrepreneurial ecosystem. Even though our sample included institutions of different sizes and profiles (see “Appendix A”) and from 11 (out of 16) federal states, contextual bias cannot be ruled out. Therefore, our results may not be transferable to other contexts, and thus, we call for replication studies to apply the developed research model in other countries, as for instance in developing nations or countries with different entrepreneurial ecosystems’ structures, as for instance where science and technology parks are a central element. This shall enable cross-country comparisons, reflecting different realities with other cultural and economic components.

Furthermore, our self-report measures might have been influenced by social desirability bias, and future studies might therefore opt to combine these with secondary data sources on key performance indicators associated with HEIs’ third mission. Specifically, studies with larger samples might apply such indicators as moderators to produce novel insights that improve our understanding of the phenomenon and raise new implications that support HEIs’ strategy and management practices.

We conclude the research agenda joining a recent wave of calls for researchers to explore further the diverse roles that DCs could play on HEIs management practices (Leih and Teece 2016; Schoemaker et al. 2018; Yuan et al. 2018; Guerrero et al. 2020). An interesting research avenue regards the role of DCs on HEIs’ strategic and digital transformation (Guerrero et al. 2020). In this sense, we propose studies on the intersection of digital economy and third mission advancements (e.g. on technology transfer, university spin-offs, entrepreneurship education and Triple Helix co-creation processes), in special considering the COVID-19 world pandemic influence on HEIs digital transformation and the reconfiguration outcomes by those with stronger/weaker DCs.

## Conclusion

This study’s findings illustrate the central role of HEI leaders in the process of producing and leveraging DCs for envisioning and advancing their institutions’ third mission. It might also pave the way for a more open discussion on institutional and policy levels about the necessary governance structures, management practices and entrepreneurial mindset required to lead HEIs into the twenty-first century.

## Appendix A

See the Table 6.

**Table 6:** Sample profile

Sample profile (n = 45)	%
Institution type	
Research University	17.8
Technical University	11.1
(Technical) University of Applied Sciences	64.4
College of Arts/Music	2.2
Other	4.4
Institution holder	
Public	95.6
Private	4.4
Location (Federal State in Germany)	
Baden-Württemberg	26.7
Bavaria	22.2
North Rhine-Westphalia	11.1
Saxony	8.8
Hessen	6.7
Lower Saxony	6.7
Brandenburg	4.4
Rhineland-Palatinate	4.4
Saxony-Anhalt	4.4
Schleswig–Holstein	2.2
Hamburg	2.2
Institution size (based on number of enrolled students)	
Less than 5.000	33.3
5.000–9.999	31.1
10.000–14.999	13.3
15.000–19.999	13.3
20.000–39.999	6.7
40.000 or more	4.4
The HEI possess a/an…	
Institute or Department for Entrepreneurship	28.8
Entrepreneurship Center	73.3
Office for Technology Transfer and/or Industry Relations	75.6
Vice-president for Entrepreneurship, Business, Industry Relations or Third-Mission	53.3
Office for HEIs Strategic Advancement (*Hochschulentwicklung*) or equivalent	35.6
Startup Acceleration Program	22.2
Startup Incubation Program	48.9
Maker Space	40.0
Living Lab	20.0
Competition/Award for Startup/Business Ideas	37.8
Seed or Venture Capital (fund, program)	6.7
Alumni Association	57.8
Number of entrepreneurship/innovation professors	
Zero	13.3
Only one	15.5
2–5	51.1
6–9	8.8
10 or more	4.4
No Answer	6.7
Approximated number of students trained in entrepreneurship per semester	
Less than 100	15.6
100–499	35.6
500–999	13.3
1000–1999	2.2
2000 or more	4.4
No answer	28.9
Approximated total number of startups already graduated from incubation program (spin-offs)	
Zero	8.9
1–9	28.9
10–49	40.0
50–99	6.7
100 or more	8.9
No answer	26.7
Approximated number of active partners from the regional ecosystem (third-mission activities)	
Less than 10	13.3
10–49	31.1
50–99	26.7
100 or more	8.9
No answer	20.0

## Appendix B

See the Table 7.

**Table 7:** Discriminant validity: indicators loading and cross-loading

	3rd Mission advancement	Dynamic capabilities	Leadership	Vision and goals
TM1_1	0.901	0.486	0.503	0.592
TMA_2	0.931	0.535	0.680	0.631
L_1	0.513	0.519	0.790	0.468
L_2	0.425	0.489	0.768	0.471
L_3	0.452	0.426	0.808	0.501
L_4	0.514	0.557	0.837	0.507
L_5	0.590	0.619	0.818	0.609
L_6	0.586	0.536	0.753	0.610
L_7	0.495	0.506	0.807	0.505
L_8	0.541	0.594	0.790	0.551
L_9	0.542	0.589	0.791	0.494
L_10	0.507	0.524	0.793	0.514
L_11	0.526	0.559	0.821	0.546
DC_1	0.489	0.731	0.601	0.572
DC_2	0.451	0.831	0.589	0.554
DC_3	0.354	0.708	0.307	0.317
DC_4	0.585	0.743	0.512	0.550
DC_5	0.615	0.816	0.572	0.682
DC_6	0.224	0.703	0.379	0.405
DC_7	0.446	0.856	0.629	0.759
DC_8	0.202	0.732	0.503	0.480
DC_9	0.305	0.755	0.448	0.567
VG_1	0.637	0.561	0.582	0.844
VG_2	0.564	0.384	0.431	0.779
VG_3	0.512	0.693	0.587	0.909
VG_4	0.508	0.754	0.574	0.778

## Appendix C

See the Table 8.

**Table 8:** Structure model predictive power

	RMSE (PLS analysis)	RMSE (linear regression)
TM1_1	1.040	1.221
TMA_2	0.845	0.903
L_1	1.388	1.476
L_2	1.352	1.531
L_3	1.405	1.644
L_4	1.273	1.522
L_5	1.403	1.635
L_6	1.107	1.302
L_7	1.365	1.552
L_8	1.102	1.327
L_9	1.501	1.785
L_10	1.273	1.497
L_11	1.432	1.416
VG_1	1.269	1.398
VG_2	1.479	1.554
VG_3	1.227	1.291
VG_4	1.180	1.351

## Footnotes

1 HEInnovate (https://heinnovate.eu), UIIN (https://uiin.org), THA (https://www.triplehelixassociation.org), HRK (https://hrk.de) and DenkFabrik (https://www.denkfabrik-he.org).
